# The Impact of Acute Stress Physiology on Skilled Motor Performance: Implications for Policing

**DOI:** 10.3389/fpsyg.2019.02501

**Published:** 2019-11-07

**Authors:** G. S. Anderson, P. M. Di Nota, G. A. S. Metz, J. P. Andersen

**Affiliations:** ^1^Office of Applied Research and Graduate Studies, Justice Institute of British Columbia, New Westminster, BC, Canada; ^2^Department of Psychology, University of Toronto Mississauga, Mississauga, ON, Canada; ^3^Department of Neuroscience, University of Lethbridge, Lethbridge, AB, Canada

**Keywords:** police, stress response, cortisol, hypothalamic-pituitary-adrenal axis, motor control, movement, muscle tension

## Abstract

Investigations of police performance during acutely stressful situations have primarily focused on higher-order cognitive processes like attention, affect or emotion and decision-making, and the behavioral outcomes of these processes, such as errors in lethal force. However, behavioral outcomes in policing must be understood as a combination of both higher-order processes and the physical execution of motor skills. What is missing from extant police literature is an understanding of how physiological responses to acute stress contribute to observed decrements in skilled motor performance at the neuromuscular level. The purpose of the current paper is to fill this knowledge gap in the following ways: (1) review scientific evidence for the physiological (i.e., autonomic, endocrine, and musculoskeletal) responses to acutely stressful exposures and their influence on skilled motor performance in both human and animal models, (2) review applied evidence on occupationally relevant stress physiology and observed motor decrements in performance among police, and (3) discuss the implications of stress physiology for police training and identify future directions for applied researchers. Evidence is compelling that skill decay is inevitable under high levels of acute stress; however, robust evidence-informed training practices can help mitigate this decay and contribute to officer safety.

## Introduction

High level motor performance skills, including the use of physical control techniques and weapons, are expected of law enforcement officers who routinely operate under acutely stressful work conditions. Mistakes in the application of physical skills can have devastating effects on the individuals involved in such incidents, with serious implications for long-term physical and mental health. Yet, much of the applied research on police performance under stress does not speak to the effects of acute stress physiology on the physical (i.e., neuromuscular) aspects of skilled motor performance, which is necessary to optimize training and best practices. The goal of this paper is to synthesize information from peer-reviewed indexed journal articles in the basic and applied sciences, which address the impact of stress physiology on skilled motor performance among police as a novel contribution to the literature. Following a brief discussion on the definition of “stress,” this paper reviews various physiological responses to stress. Using both animal and human evidence, we outline the impact of stress physiology on motor unit recruitment and the skilled performance of motoric workplace tasks. Converging lines of evidence provide a theoretical framework for understanding stress-induced decrements in skilled occupational performance, including a limited body of applied research on stress physiology among police. We conclude with a review of the efficacy for scenario-based training in improving performance decrements among police by way of adaptively inducing skill-appropriate stress and provide future directions for applied research.

## Physiological Responses to Acute Stress Exposures and Their Influence on Skilled Motor Performance

### Defining Stress

The term “stress,” as originally introduced by Selye (1956), refers to a challenge (i.e., stimulus), psychological or physical in nature, that threatens (or that is perceived to threaten) the internal balance or homeostasis of physiologic systems. However, stress has been used interchangeably to describe the physiologic stress response itself, the stimulus-response interaction, or even the whole spectrum of interacting factors (e.g., stimulus, cognitive appraisal, perception, and coping style) (Violanti and Aron, 1995; Anshel et al., 1997; Anderson et al., 2002; McEwen, 2008). For clarity, in the current review, stress is defined according to the definition provided by Selye (1956), as a challenge or stimulus and is separate from the physiological reactivity that follows in response to the stress or challenge. Thus, a stressor is anything that leads to physiological stress reactivity. As such, stressors can be “real” externally perceived objects such as a weapon, or internally perceived appraisals of an uncertain or potentially threatening situation. The intensity of the physiological reaction to a stressor is therefore highly individual and situationally dependent (Dewe, 1993; Peters et al., 1998). Many variables, including personal attributes, implicit or explicit neurocognitive appraisals, coping strategies, social support, and past experiences may modify the physiological stress response in any given situation and can account for the different response of two individuals exposed to the same stressor (Anshel et al., 1997; Anderson et al., 2002; Babenko et al., 2015; Ambeskovic et al., 2017).

Independent of the type of stressor, a physiological stress response is initiated by the brain and can be understood as a nonselective response (i.e., fight-or-flight response) that acts to reorient the individual’s cognitive and physiologic capacities to deal with the challenge and return to the original state of homeostasis (Chrousos and Gold, 1992). Situations or stimuli that are novel, unexpected, or unpredictable are generally perceived as more stressful and associated with a stronger physiological response (Thayer and Sternberg, 2006). The following sections will review key physiological systems that respond to acute stressors in order to provide a theoretical context for understanding how occupationally relevant stress can result in decrements to skilled motor performance.

### Physiological Responses to Acute Stress Exposure

#### The Autonomic Nervous System Response to Stress

The physiological response to a stressor occurs largely outside conscious awareness, arising from neural circuits that are also responsible for imbuing meaning and personal relevance to external stimuli (LeDoux and Pine, 2016; Ginty et al., 2017). In response to a stressor, a complex cascade of internal processes is stimulated and coordinated by the brain, beginning with autonomic nervous system (ANS) activation, thermogenesis, elevated blood glucose levels, and other responses in the service of returning the body to homeostasis (e.g., see Rivolta et al., 2014). The ANS regulates the function of internal organs, such as respiratory and cardiovascular activation (DeRijk and de Kloet, 2005; Agorastos et al., 2018). The ANS is divided into sympathetic (SNS) and parasympathetic (PNS) branches (Thayer and Sternberg, 2006). Although the relationship between SNS and PNS activity is complex and should not be thought of as an “either/or” system, it is generally accepted that during a physiological stress response, the SNS is activated and PNS, responsible for calming and stabilizing the body, is withdrawn (Thayer and Sternberg, 2006). The degree of a SNS response is thought to be determined by one’s perception of how threatening the stimulus is, even if the perception is not within conscious awareness (Kalisch et al., 2015; LeDoux and Pine, 2016). Further, the physiological responses during stress can be enhanced or diminished by psychological factors, such as perceived control over the situation (McEwen, 2008).

Adaptive SNS arousal is beneficial for performing optimally during critical incidents. The benefits of adaptive arousal include alertness, focused attention, and improved cognitive performance (Jamieson et al., 2010). During adaptive SNS arousal, sensory perception including visual, auditory, and olfactory senses are enhanced (McNish and Davis, 1997). Improved sensory awareness increases an individual’s ability to successfully address a stressor (Kalisch et al., 2015). However, during more extreme stress, the SNS response may dominate or become maladaptive and performance starts to deteriorate. The mechanism by which the autonomic stress response influences skilled motor performance is poorly understood in humans, and animal models have been used to help explain the underlying mechanisms. Evidence for skilled motor performance decay in animal models and various occupations are reviewed below, with emphasis on police.

#### Neuroendocrine Stress Model: The Brain’s Hormonal Response to Threat

If a stressor does not dissipate immediately, the brain will initiate an endocrine response following the activation of the ANS. The endocrine response begins *via* the activation of the hypothalamic-pituitary-adrenal (HPA) axis. The HPA axis prompts the release of hormones, including corticotropin releasing factor (CRF) from the hypothalamus (DeRijk and de Kloet, 2005; Frasch et al., 2018). CRF in turn triggers the release of adrenocorticotropic hormone (ACTH) from the anterior pituitary, which then reaches the cortex of the adrenal glands to stimulate secretion of adrenal glucocorticoids (GCs). The increase of adrenal GCs in blood, such as cortisol in humans and corticosterone in rodents (in the following both referred to as CORT), is considered the main physiological correlate of stress (de Kloet et al., 1998). GCs can travel with the blood to reach any organ in the body, including crossing the blood-brain barrier to exert their most prominent actions on the brain (Chrousos, 2009). The action of GCs has been explained by the concept of the “inverted U-shape” (Sapolsky, 1997; Salehi et al., 2010; see also Di Nota and Huhta, 2019). Depending on the duration and severity of the stress, the response to GCs can promote or impede brain function and adaptive behaviors. Thus, optimal GC levels ensure best performance in day-to-day work situations, including acutely stressful policing situations (Regehr et al., 2008).

##### Repeated Exposure to Acute Stress: Causes and Effects

While this paper addresses acute exposures to stress, the repeated and prolonged exposure to occupational stress in police officers can manifest as chronic stress, and this in turn impacts skilled performance under acutely stressful events. In the brain and other organs, CORT binds to two types of receptors – the high-affinity mineralocorticoid receptor (MR) and the low-affinity glucocorticoid receptor (GR; de Kloet et al., 1998). Once bound, CORT and GR build a complex that binds to DNA to activate gene expression and generate lasting changes in cellular functions. Furthermore, GRs within the HPA axis serve an important negative feedback function, like a thermostat, to downregulate GC production once the stressful event subsides. The negative feedback regulation is critical to end the stress response when dealing with an acute, short-term stress. The enduring activation of the stress response, however, when faced by lasting, chronic stress, may eventually be associated with down-regulation of GRs (Mizoguchi et al., 2003) or the development of GR resistance (Cohen et al., 2012) at the feedback sites of the brain. This change will diminish the negative feedback regulation by the HPA axis and result in prolonged maintenance of the stress response and prolonged exposure of the brain to higher levels of GCs. Extended periods of stress and elevated levels of GCs can alter brain function and increase vulnerability to neurological disease (McEwen, 2000; Madrigal et al., 2003; Cottrell and Seckl, 2009), including those conditions that affect motor function. Recent evidence shows consistently elevated levels of diurnal (i.e., daily fluctuating) CORT among police officers relative to the general population, with specialized tactical officers demonstrating even higher levels of resting CORT than frontline officers (Planche et al., 2019). These findings provide a physiological basis for long-term risk of physical and mental disorders among law enforcement personnel (Franke et al., 2002; Ramey et al., 2009; Joseph et al., 2010; Violanti et al., 2018), which may be further compounded by the level of risk exposure in policing subspecialties (Planche et al., 2019).

##### Manifestation of Chronic Stress in the Musculoskeletal System

It has long been hypothesized that increased muscle tension occurs during stressful situations (Malmo et al., 1951), although the data are mixed and depend on the population tested. Westgaard and Bjørklund (1987) demonstrated a consistent pattern of increased muscle tension over and above that required to maintain postural stability during psychophysiological testing. In a series of studies, Lundberg et al. (1994) reported increased muscle tension above that required to overcome the physical load of cashier work when psychological stress was induced and also demonstrated a link between trapezius myalgia and increased stress (both self-report and increased blood pressure) in cashiers (Lundberg et al., 1999). Lundberg (2002) concluded that both physical (such as repetitive movement) and psychosocial (such as increased mental stress) work may be related to the development of upper extremity disorders. In a prospective study, Veiersted (1994) showed higher “resting tension” in the trapezius muscles of chocolate packers who became symptomatic with shoulder pain when compared to healthy controls. Lundberg et al. (1994) suggest psychological stress, with or without physical load, may play a role in musculoskeletal disorders by increasing muscle tension through activation of low threshold motor units creating overload with subsequent degenerative processes.

While Anderson et al. (2002) have demonstrated anticipatory and reactive stress responses in police officers across entire shifts, it can be expected that increased muscle tension may play a role in the physical response patterns and efficiency of police officer movement. As skilled performance activates muscle fibers in a pattern from low to high threshold motor units, it can be hypothesized that chronic stress could shift both the recruitment pattern (requiring more higher threshold motor units to be active) and efficiency of movement (requiring more force to overcome the resting tension) in both stressed and non-stressed environments. However, direct measurement of neuromuscular activation in police officers has yet to be conducted during acutely stressful situations (i.e., real-world or simulated critical incidents), or following prolonged exposure to stress. In a study on combat soldiers, Tornero-Aguilera et al. (2017) found greater lower body muscle strength (measured by various vertical jumps) and higher lactate concentrations in elite relative to novice soldiers following stressful combat scenarios, suggesting that experience (and training) contributes to greater efficiency of movement. In a study by Lewinski et al. (2015b), officers performed several sprinting maneuvers with and without their heavy tactical and safety equipment (approximately 9 kg, or 11% of the participants’ body mass). Officer velocity and acceleration was significantly reduced by the additional weight of the equipment, which is required for all officers. These results demonstrate a significant physical burden on officers’ ability to operate and further underscore the need to investigate neuromuscular aspects of police performance.

### Skilled Motor Impairments in Response to Acute Stress

Even popular culture recognizes the impact of acute stress on motor performance; as illustrated by a classic movie situation in which someone is stalked by an aggressor, gross motor behavior like running away is not problematic. But once at the door, they frantically try to unlock it and experience difficulty inserting the key into the lock. This example best exemplifies the considerable impact of stress on motor skills that require finer control over developed muscle tension, and muscle fiber firing patterns that allow for smooth and accurate movement.

As opposed to skilled movements requiring finer motor control, general motor patterns, such as navigating through a novel environment, are determined mainly by cognitive, emotional, and motivational aspects, and therefore they do not offer a useful model for studying the mechanisms of how stress interacts with the motor system. Skilled movements, however, are an ideal model to examine discrete motor disturbances in response to stress and stress-induced neuroplasticity. Studies in rats have shown that, through direct interaction of CORT and GRs as well as emotional changes, stress can alter the movement trajectories and accuracy of skilled movements in forelimbs and hindlimbs, and disrupt inter-limb coordination (Metz et al., 2005). Furthermore, stress perturbs postural stability in both human subjects and rodents (Maki and McIlroy, 1995; Metz et al., 2003).

A variety of studies have described the contribution of distinct motor areas to skilled reaching movements in humans and animals, mainly through the study of focal lesions of the underlying neural substrate. These investigations revealed discrete changes in the qualitative movement trajectories of reaching for food produced by areas such as the red nucleus (Morris et al., 2011), corticospinal tract (Metz and Whishaw, 2002), motor cortex (Kirkland et al., 2012), striatum (Faraji and Metz, 2007; Jadavji and Metz, 2008; Faraji et al., 2014), and cerebellum (Azim et al., 2014). In these systems, chronic stress was shown to diminish the ability to perform rotatory limb movements in skilled reaching and reduce movement accuracy in the forelimb subsequently leading to significant reduction in skilled reaching success (Metz et al., 2005; Metz, 2007). Furthermore, this study showed that both stress and CORT affect the temporal aspects of movement in that reaching movements became faster and more frantic. This finding concurs with the notion of the fight-or-flight response, which proposes that an individual mobilizes energy to move faster and escape from a threatening situation. Using detailed frame-by-frame analysis of video recordings, the authors suggested that this gain in function may come at the expense of movement accuracy, which in the animal studies was illustrated by animals needing more attempts to grasp a single pellet in parallel to diminished reaching success (Metz et al., 2005; Kirkland et al., 2012). Such a failure to maintain success rates under stressful conditions might also reflect altered sensory feedback, such as loss of haptic feedback and inability to adjust the paw position to grasp a food pellet. Furthermore, dependent on the task, stress and CORT may also impede the use of compensatory movement strategies, such as the modification of a movement strategy to adjust the paw trajectory in a task-specific manner after brain damage (Kirkland et al., 2012).

#### Central Mechanisms of Skilled Motor Performance Decay

The physiological mechanisms that predict skilled motor performance decay under high stress primarily involve the brain’s ability to initiate the proper motor sequences and less to do with changes at the contractile sites within muscle tissue. In support, there are three lines of evidence rooted in fundamental scientific research in animal and human models that suggest that stress and GCs are likely to affect central motor control.

1. *Brain regions participating in motor control have receptors for GCs*. Brain regions participating in motor control, such as motor cortex, cerebellum, basal ganglia, and spinal cord, have a significant density of GRs (Ahima and Harlan, 1990; Ahima et al., 1991; Marlier et al., 1995) rendering them susceptible to the influence of stress-induced elevations of GCs. Accordingly, stress can change the execution of skilled movements. For example, stress and GC treatment significantly diminish skilled reaching success when reaching for a food pellet and impair limb placement accuracy in skilled walking on a horizontal ladder (Metz et al., 2005; Merrett et al., 2010; Kirkland et al., 2012). Stress hormones including CORT, CRF, and ACTH may interfere with motor function *via* direct interactions with GRs, but may also interact with the brain’s catecholaminergic systems (Finlay et al., 1995), in particular dopamine, which critically controls balance and fine motor function (Abercrombie et al., 1989; Smith et al., 2008).
2. *Stress hormones can modify pathological processes of the motor system.* Stress is a primary candidate to interact with pathological processes and recovery after motor system lesion. For instance, work investigating rat models has shown that stress can slow motor recovery after dopamine depletion lesion (Smith et al., 2008; Hao et al., 2017) or motor cortex lesion (Metz and Whishaw, 2002; Faraji et al., 2011; Kirkland et al., 2012). The reduced functional recovery in stress-treated animals seems to parallel with increases in lesion size (Kirkland et al., 2008). Stress might exert these effects in association with altered neurotrophic factor expression (Sun et al., 2014). In human studies, stress has also been recognized as possibly the single most important factor to affect recovery and neuroplasticity after motor system injury (Walker et al., 2014).
3. *Stress can modulate motor patterns by altering the affective state*. In addition to direct effects of stress hormones on nervous system function, stress also alters the emotional state which in turn modulates general motor patterns (e.g., walking) and fine movements (e.g., reaching and grasping: Lepicard et al., 2003; Metz et al., 2005) and balance (Maki and McIlroy, 1995). Thus, stress-induced anxiety can modulate motor behaviors, such as motor activity in open field tests (Treit and Fundytus, 1988; Zhu et al., 2014; Monteiro et al., 2015). The relationship between stress-associated anxiety and decrements in motor performance is further supported by the observation that mouse strains bred for high anxiety traits show greater motor skill impairments than less anxious mouse strains (Lepicard et al., 2000, 2003). These effects can be reversed by treatment with anxiolytic drugs, which were shown to improve motor skill (Lepicard et al., 2003; Metz et al., 2003).

Patterns of maladaptive motor strategies have been observed in police officers, which interact with perceived stress and impact skilled motor performance. By inhibiting a preferred movement strategy (i.e., step then fire) in experienced police officers, self-reported anxiety increased and shooting accuracy was reduced, even if the preferred movement strategy puts the officer at greater risk (Nieuwenhuys et al., 2017).

## Police Stress Physiology and Skilled Motor Performance

While the influences of acute stress on police performance have largely focused on cognitive functions, such as decision-making, learning, and memory (see Morgan III et al., 2006; Taverniers et al., 2013; Hope, 2016), a comparably small number of studies have investigated the influence of stress physiology on skilled motor performance. The following will summarize the comparably small number of applied studies that have focused on the influence of stress physiology on skilled motor performance among police.

### Stress Physiology During Active Duty and Training Among Police

Individuals employed in public safety and protection environments are often exposed to a wide variety of stressors, and the response to each type of stressor will vary greatly across the population or different responders. As one would expect, various physiological measurements have revealed increased stress reactivity during physical and psycho-social aspects of police work in active duty settings (Anderson et al., 2002; Andersen et al., 2016b,c). Anderson et al. (2002) found increased cardiovascular stress responses while measuring police officers during periods of heightened physical demand (e.g., escalating use of force activities), situations where there is potential threat (e.g., hand on gun situations) and during periods of anticipation (e.g., pre-deployment or driving to an event with lights and sirens). These data are consistent with those of Anshel et al. (1997) who rated facing unpredictable situations as the most severe acute stressor experienced by this population and which is characteristic of most police deployments.

More recently, work by Baldwin et al. (2019) measured officers’ cardiovascular activity (peak heart rate above resting average) and physical movement (speed) to reveal unique and dynamic influences of physical and psychological stressors during various phases of general duty calls (i.e., arriving on scene, encountering a subject). Officers’ stress responses increased with the priority of a call (i.e., very urgent > urgent > routine) and with the report of a weapon present. Calls involving use of force had elevated stress responses during all phases of the call, including dispatch, en route, and arrival. Officer age, gender, years of experience, and level of specialized operational skills training in use of force and other physical tactics did not significantly modulate stress reactivity (Baldwin et al., 2019).

A misconception remains among some police trainers and law enforcement agencies that simulation training cannot elicit real world stress responses because the results of the training are not grievous. A growing body of research shows that this is not the case and demonstrates significant increases in police officers’ stress physiology during high threat simulations or scenarios relative to rest (Oudejans, 2008; Nieuwenhuys and Oudejans, 2010, 2011; Nieuwenhuys et al., 2012, 2015; Andersen and Gustafsberg, 2016; Andersen et al., 2016a, 2018; Arble et al., 2019; Bertilsson et al., 2019b). Using salivary (Andersen et al., 2016a) and cardiovascular biomarkers of stress reactivity (Oudejans, 2008; Nieuwenhuys and Oudejans, 2010, 2011; Nieuwenhuys et al., 2012, 2015), researchers have demonstrated significant increases in stress reactivity during high-threat relative to non-violent or low-threat scenarios. During virtual lethal use of force training, Groer et al. (2010) demonstrate high levels of stress reactivity through several different endocrine measures, concluding that training scenarios designed and delivered in a virtual modality were capable of producing realistic physiological stress responses (see section “Implications for Motor Skills Training” for discussion of performance gains with virtual training).

Armstrong et al. (2014), Andersen and Gustafsberg (2016), and Andersen et al. (2018) also show ecologically valid increases in heart rate among police officers when accessing weapons and engaging in live-action use of force training scenarios. Recent evidence from Bertilsson et al. (2019b) show that repeated and consecutive performance of stressful training scenarios lead to cumulative physiological effects, such that increases in heart rate are observed even prior to scenario onset (i.e., anticipatory stress) and continue to escalate with subsequent stressful exposures. Additionally, Bertilsson et al. (2019b) demonstrate a complex pattern of pupil dilation that also reflects sympathetic stress responsivity, with greater increases in pupil dilation during early tasks. These findings bear greatly on the immediate and cumulative impact of repeated exposure to stressful encounters on police physiology, but performance results were not reported. Direct comparison of training and general duty stress physiology by Andersen et al. (2016c) confirm that scenario-based training can successfully reproduce the stress reactivity one would expect in real-world environments.

### Stress-Induced Decrements in Skilled Motor Performance Among Police

Physiological responses to stressful encounters observed in police officers and described above are typically paired with decrements in skilled motor performance. The investigations by Nieuwenhuys, Oudejans, and colleagues summarized above demonstrate significantly greater physiological arousal as well as shooting skill deterioration under high stress conditions that included a heavily armed person or cannon that could shoot back at the officer, versus low threat conditions with static or unarmed targets. Stress-induced decrements to participants’ shooting performance include decreased accuracy, faster reaction times, and more false positives (i.e., shooting unarmed targets) (Oudejans, 2008; Nieuwenhuys and Oudejans, 2010, 2011; Nieuwenhuys et al., 2012, 2015). Together, these results demonstrate significant skill deterioration during stressful shooting situations that feature either physical or psychological stress.

As another variant of gross (versus fine) motor skills, complex motor skills typically used in the line of duty, including non-verbal communication, arrest, and self-defense skills, have also been shown to degrade under high versus low stress conditions (Nieuwenhuys et al., 2009). Arble et al. (2019) show differential effects of cardiovascular stress responses on motor skill decrements such that increased antithrombin – an anti-clotting factor released following increases in heart rate – predicted improved overall performance as evaluated by experienced police trainers, but also predicted a specific deficit in verbal communication. Nieuwenhuys and Oudejans (2010) measured shooting accuracy, movement times, head orientation, and blink behavior in police officers in a low anxiety condition with a non-threatening opponent and in a high anxiety condition against a threatening opponent who could shoot back using colored soap cartridges. Under the high anxiety condition, participants changed their body position, acted faster, and increased blink frequency, hence increasing the amount of time the eyes were closed. As a result of quicker movement, a compressed body position that might not facilitate proper shooting mechanics and an extended period of time without visual contact with the suspect (i.e., more blinking), significant decrements in shooting performance were observed. Similarly, Renden et al. (2014) found police officers were “less able to inhibit stimulus-driven processing (fear of getting hit) and enforce goal-directed processing (perform the skill as well as possible) leading to avoidance behavior (p. 100)” with a decrease in simulated arrest performance (Renden et al., 2014). Officers’ reactions to stressful encounters are also bound by their natural (i.e., untrained) startle responses, which induce muscle contractions within milliseconds of perceiving a threatening stimulus. Two investigations by Lewinski et al. (2013, 2015a) systematically evaluated officer reaction times and skilled motor responses from various tactical starting positions. Kinematic analyses of officer movement patterns reveal that officers who successfully completed retreat movements *before* drawing their firearms were faster at reaching a safe zone, and that officer’s finger-indexing (e.g., high on the slide) and tactical positions (e.g., low-ready, high-guard) significantly impacted performance time. Therefore, training that promotes skill learning by inducing stress can help override natural, unconditioned startle responses or preferred positioning that could endanger officers during critical incidents.

These investigations provide evidence for decrements in the cognitive (i.e., stimulus versus goal) and visuomotor processes (i.e., attention, perception) underlying motor performance under stressful simulated environments. However, the precise mechanisms by which occupationally relevant stress physiology impairs neuromuscular aspects of skilled motor performance among law enforcement remain unknown. Inferences from fundamental scientific literature using animal and human models described above can be drawn, but future investigations accounting for the unique and highly dynamic nature of police work can begin to fill this gap in the applied literature while also contributing to the development of evidence-based training practices.

## Implications for Motor Skills Training

Prior to skill mastery, novice learners need to learn fundamental motor skills in the absence of high stress conditions. Component “chunks” of behavior should be learned in a progressive manner, with increasing skill complexity once movement patterns are engrained, efficient, and accurate. Once primary skills can be demonstrated at a high level of performance, training should progress to the application of these skills in a wider variety of complex situations that also increase the stress response (*cf*, Di Nota and Huhta, 2019). While the underlying mechanisms remain unknown, Vickers and Lewinski (2012) provide evidence for experience- and (possibly) training-related differences between novice and elite police officers in visuomotor (gaze control) and motor performance during stressful live training scenarios. Officers with more experience made fewer decision-making errors, shot more accurately, had faster motor onsets (i.e., draw/aim/fire) and greater visual fixation on targets (referred to as “quiet eye”) prior to firing, contributing to greater accuracy and fewer errors. Highlighting specific motor decrements in novice versus experienced officers can help inform the skills that need to be targeted in firearm and tactical training, especially for recruits.

A growing body of research reveals the effectiveness of live simulation or scenario-based training for improving stress-induced decrements to skilled motor performance among first responders. Scenario-based training is founded on adult learning principles that adaptively expose officers to realistic and occupationally relevant stressors in a safe, controlled environment. With constructive feedback from experienced trainers, correct motor strategies are encoded either through correct performance or feedback on errors that can be made without life-threatening consequences. Scenario-based training is effective for teaching both new skills or refreshing essential skills that can be recalled and utilized more successfully under the same stressful conditions with which they were taught (Murray, 2005; Barney and Shea, 2007; McNaughton et al., 2008).

Physiological stress responses are induced during scenario-based training by using realistic actors, environments, props, and subject matter that are challenging for the skill level and experience of the student (Birzer and Tannehill, 2001; Murray, 2006; Bennell et al., 2007; Krameddine and Silverstone, 2015; Andersen and Gustafsberg, 2016; Andersen et al., 2018). Scenario content should be based on situations and events that the trainee will typically face in their day-to-day duties, but may also include preparation for worst-case events. However, worst-case events are rare and research indicates that a sole focus on such events in training may result in an individual overweighting the probability of rare events at the expense of learning how to manage the most common and realistic exposures they will face in their general duties (Birzer and Tannehill, 2001; Harman and Gonzalez, 2015; Harman et al., 2019).

In the previously reported investigations that show stress-induced decrements to skilled police performance, officers that trained with live opponents under high threat, stress-inducing conditions performed better than officers trained using static targets under low threat conditions (Oudejans, 2008; Nieuwenhuys and Oudejans, 2011). Andersen and colleagues have successfully improved police lethal use of force decision-making following an intervention that targets the modulation of the physiological stress response during training (Andersen and Gustafsberg, 2016; Andersen et al., 2018). Further, significant improvements in an occupationally relevant health indicator, specifically, recovery from stress, as measured by heart rate returning from maximum to rest following critical incident scenarios, were maintained up to 18-months of follow-up (Andersen et al., 2018). Similar to the existing evidence for stress-induced decrements to skilled police performance, training-induced improvements to performance mainly pertain to decision-making (i.e., shoot/no-shoot) and visuomotor processes underlying perception and attention (i.e., gaze, blinking). Future investigations that directly measure training-related neuromuscular gains including muscle force, speed, and resulting behavioral accuracy are required to fill gaps in the applied police literature regarding skilled motor performance under stress.

The use of video simulation technologies for police use of force training is increasing in popularity (Davies, 2015). The stated benefits of this approach include controlling for dynamic situational factors such as actor behavior, which may vary in live simulations despite pre-defined scripts and situational outcomes (see Bennell et al., 2007). The uptake in usage is surprising given the lack of research validating the effectiveness of virtual simulation training that has been used for various purposes, including lethal use of force decision-making and shooting accuracy. Nieuwenhuys et al. (2015) failed to show improvements in high-stress performance decrements following training with video scenarios, while their interventions with stressful live scenario training did show significant post-training performance improvements (Nieuwenhuys and Oudejans, 2011). Forthcoming evidence demonstrates significantly higher errors in lethal force decision-making and significantly less autonomic stress arousal during video simulation trials compared to live-actor simulation trials within the same sample of frontline police officers (Di Nota, Boychuk, Andersen, under review). More empirical research is needed on a variety of virtual simulation tools and platforms before considerable investments are made in technologies and training methods that are less effective than live scenario-based training, and in the worst case could reinforce maladaptive (i.e., under-reactive) stress responses and errors in both cognitive decision-making strategies and tactical motor skills.

### Future Directions for Applied Research on Motor Performance

While animal models would suggest skilled motor performance decrements are inevitable under high stress, applied police research has shown significant improvements in various aspects of skilled motor performance following training under highly stressful conditions. Nonetheless, the direct influence of occupationally relevant stress physiology on neuromuscular aspects of skilled motor performance among law enforcement remains less clear. Before such investigations can occur, it is important that researchers carefully evaluate and define “performance” on more dimensions than just the behavioral outcome (i.e., shoot or no-shoot), which can be interpreted as reflecting cognitive decision-making processes as much as physical performance capability, quality, or success (see Renden et al., 2017). Haller et al. (2014) evaluated officer performance according to several aspects that together comprised an “ethological profile” (p. 3) at different stages of critical incident scenarios. A recent investigation by Bertilsson et al. (2019a) also evaluated multiple performance categories, including general motor control distinct from motor control of voice, verbal content, as well as spatial and temporal tactical implementation. By scoring verbal, orientation, positional, and movement parameters, training and evaluation become more detailed by providing feedback on specific elements of officer’s behaviors that might be maladaptive (e.g., poor positioning) but not contribute to a negative outcome per se (were not injured by the suspect in this scenario, but could put themselves in danger in future situations). This study is a step in the right direction and utilizes objective physiological measures of individual stress responsivity to demonstrate a link between decrements in skilled motor performance (i.e., complex verbal and physical de-escalation) and stressful occupationally relevant exposures among police.

Once skilled motor performance has been operationalized, various non-invasive methodological approaches can be used to pinpoint the contribution of stress physiology on musculoskeletal functioning. Electrical brain signals originating in motor cortex and terminating in targeted muscle groups can be measured using surface-level electrodes *via* electroencephalography (EEG) and electromyography (EMG), respectively. The frequency (time series) and amplitude of neuromuscular signals under high- and low-stress conditions could be compared to identify the influence of stress on the speed and timing of motor skills, including reaching for and utilizing a firearm. Muscle tension as recorded by EMG could also reveal subtle differences between anticipatory (i.e., psychological) and physical stress, clarifying whether these stressors manifest similarly in musculoskeletal architecture.

Training aimed at reducing police performance errors in the use of lethal force have already started to focus on physiological-based interventions, including physiological stress modulation (Andersen and Gustafsberg, 2016; Andersen et al., 2018). In an exploratory pilot study, Johnson et al. (2014) compared performance (i.e., shoot/no-shoot decision-making), heart rate, and EEG activity between experienced military and police officers and civilians during virtual use of force scenarios. As expected, performance errors were significantly higher among civilians, and EEG data showed several experience-dependent differences, including greater task-related changes in alpha power among experts, including when a shot was taken relative to rest. Further, EEG metrics differentiated intermediates (<10 years’ experience) from experts (>10 years’ experience), consistent with previous evidence for greater alpha suppression (indicative of cognitive load and task engagement) when engaging in a familiar motor task relative to less familiar experts and non-experts (Di Nota et al., 2017). Johnson et al.’s (2014) groundbreaking work suggests that non-invasive neurophysiological recordings could be a useful training tool for optimizing virtual lethal force/decision-making training, and objectively assessing skill learning and expertise. Further validation of existing training paradigms, including virtual technologies, are needed for truly evidence-based training practices.

## Conclusion

A growing body of literature has demonstrated significant physiological activation following exposure to acutely stressful events among police, both in real-world and simulated settings. However, there are little data that document the direct impact of stress physiology on skilled motor performance at the central (brain and spinal cord) or peripheral (neuromuscular) level. Future investigations in this regard can aid in further optimizing effective interventions that train adaptive physiological stress responses to acutely stressful situations and which reduce errors in lethal use of force and improve physiological recovery from stress. Together, this evidence-based line of research can immediately improve stress-induced decrements to skilled motor performance, preserve occupational and public safety, and reduce the risk for physical and mental health disorders disproportionately observed in first responder populations (Franke et al., 2002; Ramey et al., 2009; Joseph et al., 2010; Carleton et al., 2018; Violanti et al., 2018).

## Author Contributions

GA and GM completed the first conceptualization and draft of the manuscript. JA conceptualized components and wrote sections of the manuscript. PD contributed to writing significant revisions of the final manuscript.

### Conflict of Interest

The authors declare that the research was conducted in the absence of any commercial or financial relationships that could be construed as a potential conflict of interest.
